# The effects of bariatric surgery on dementia in patients with diabetes and obesity

**DOI:** 10.1002/alz.71802

**Published:** 2026-08-21

**Authors:** Erik Stenberg, Yang Cao, Björn Eliasson, Erik Näslund

**Affiliations:** ^1^ Department of Surgery, Faculty of Medicine and Health Örebro University Örebro Sweden; ^2^ Clinical Epidemiology and Biostatistics, School of Medical Sciences, Faculty of Medicine and Health Örebro University Örebro Sweden; ^3^ Unit of Integrative Epidemiology Institute of Environmental Medicine, Karolinska Institutet Stockholm Sweden; ^4^ Department of Medicine Sahlgrenska University Hospital Gothenburg Sweden; ^5^ Division of Surgery, Department of Clinical Sciences Danderyd Hospital, Karolinska Institutet Stockholm Sweden

**Keywords:** alcohol dementia, Alzheimer's disease, bariatric surgery, dementia, diabetes mellitus, epidemiology, humans, obesity, type 2, vascular dementia, weight loss

## Abstract

**INTRODUCTION:**

Type 2 diabetes (T2D) and obesity are among the most important risk factors for dementia. Although weight loss is an important preventive strategy, the impact of metabolic and bariatric surgery (MBS) on dementia risk remains contradictory.

**METHODS:**

We conducted a propensity score matched study using nationwide, high‐quality clinical registries to compare the outcomes in patients with obesity and T2D who underwent MBS with matched controls who did not undergo surgery.

**RESULTS:**

Over a mean follow‐up of 9 years, MBS was associated with a reduced risk of dementia (15‐year cumulative incidence 1.8% vs. 2.7%, subdistribution hazard ratio [SHR] 0.60, 95% CI 0.47–0.76). The incidence was lower for Alzheimer's disease (1.2% vs. 1.9%, SHR 0.56, 95% CI 0.41–0.76) and vascular dementia (0.4% vs. 0.7%, SHR 0.49, 95% CI 0.29–0.85), but increased for alcohol‐related dementia (0.2% vs. 0.04%, SHR 3.67, 95%CI 1.33–10.14).

**DISCUSSION:**

These findings suggest that the collected long‐term effects of MBS may reduce the risk of developing dementia in individuals with obesity and T2D.

## Highlights

- We examined the association between metabolic and bariatric surgery (MBS) and risk of dementia.
- Patients who underwent MBS was compared to matched individuals with type 2 diabetes (T2D) and obesity.
- The risk for dementia was reduced by 40% among patients who underwent MBS.
- Reduced risk was seen for Alzheimer's disease (AD) and vascular dementia but increased for alcohol dementia.
- MBS may reduce the risk for new‐onset dementia in patients with T2D and obesity.

## 1 BACKGROUND

The global prevalence of dementia is increasing and is estimated to further increase from 57 million in 2019 to 153 million by 2050.^1^ While the majority of the increase is driven by demographic factors of an ageing and growing population, approximately 45% of dementia cases may be due to modifiable factors, many of which may have a direct or indirect connection with obesity.^2^, ^3^ Type 2 diabetes (T2D), obesity, and smoking have been reported as the top three modifiable contributors to dementia,^4^ with obesity being reported to be the most important factor among women.^5^ Middle‐aged people with obesity have 20%–40 % higher risk of developing mild cognitive impairment and dementia later in life than those of normal weight, with increased risk with longer duration of obesity.^6^

Addressing modifiable risk factors remains an important strategy for preventing dementia. Metabolic and bariatric surgery (MBS) is known to result in a stable long‐term weight reduction, and remission or improvement of several metabolic risk factors also linked to dementia, including hypertension, dyslipidemia, and T2D.^7^ However, MBS is also associated with increased risk for depression and alcohol use disorders, which in turn are known to increase the risk for dementia.^3^ Previous studies have suggested that MBS improves vascular efficiency and cortical thickness of the temporal lobes and may improve cognition.^8^, ^9^ Through effects on glucagon‐like peptide 1 receptor agonists (GLP‐1RA) and cardiovascular protective effects, MBS may be hypothesized to reduce the incidence of Alzheimer's disease and vascular dementia.^10^

Previous studies have reported contradictory results with both reduced risk for Alzheimer's disease^11^ and an increased risk for dementia after MBS.^12^ The aim of the current study, therefore, was to evaluate the risk for dementia with a particular focus on Alzheimer's disease among patients with obesity and T2D in a matched study based on high‐quality data using validated, nationwide prospective databases.

## 2 METHODS

The study is a matched cohort study based on data from the Scandinavian Obesity Surgery Registry (SOReg) and the National Diabetes Register (NDR), including patients who underwent primary Roux‐en‐Y gastric bypass (RYGB), single anastomosis gastric bypass, or sleeve gastrectomy (SG) in Sweden from January 2007 until December 2023.

The surgical group was identified through SOReg, a national research and quality registry established in 2007 that covers virtually all MBS procedures performed in Sweden. The registry is continuously validated with high‐quality of the data.^13^ A non‐operated control groups was based on patients with T2D with registration in the NDR. The NDR began in 1996 and currently includes approximately 90% of individuals with T2D, with longitudinal follow‐up registration.^14^

**RESEARCH IN CONTEXT**

1. **Systematic review**: Studies evaluating the effects of metabolic bariatric surgery (MBS) have suggested improved cognition, but results on the risk for dementia have been contradictory.
2. **Interpretation**: In this propensity score matched study, patients with type 2 diabetes (T2D) and obesity that underwent MBS had a lower risk of all‐cause dementia, Alzheimer's disease and vascular dementia but an increased risk for alcohol‐related dementia.
3. **Future directions**: The collected long‐term effects of MBS may reduce the risk of developing dementia, further supporting the benefits of MBS among patients with T2D and obesity in particular for patients at increased risk for dementia.

Adults aged 18–75 years with obesity (body mass index [BMI] ≥ 30 kg/m^2^) were considered for inclusion.

By use of personal identification numbers (unique to all Swedish residents), the databases were linked to the National Patient Registry (for hospital admissions and outpatient contact in specialized care),^15^ the National Prescribed Drugs Registry (covering all dispensed prescribed drugs in Sweden),^16^ the longitudinal integrated database for health insurance, labour market studies (to cover socioeconomic data at an individual level),^17^ and the Total Population Registry (to cover mortality and migration).^18^

To reduce the risk of confounding by indication, patients who migrated or died during the first year after the index date, patients who received pharmacological treatment for dementia (ATC‐code N06D) or had a diagnosis of any type of dementia up until 1 year after the index date were excluded. Patients who had a previous diagnosis of cerebrovascular events (International Classification of Diseases Revision 10 [ICD‐10] codes: I60‐I64), brain cancer (ICD‐code: C71), or pharmacological treatment for Parkinson's disease or parkinsonism (ATC‐code: N04) were also excluded (Supplements‐ SAP).

### 2.1 Definition of baseline variables

Baseline variables were based on combined data from the SOReg, the NDR, the National Patient Registry, the National Prescribed Drugs Registry, and the longitudinal integrated database for health insurance, labor market studies (Table S1).

### 2.2 Outcomes

The main outcome was the new onset of all‐cause dementia as diagnosed in the National Patient Registry (ICD‐10 codes F00‐03, F10.6, F10.7, or G30) or initiation of pharmacological treatment for Alzheimer's disease (ATC‐code: N06D).^19^

Secondary outcomes were: Alzheimer's disease, which was defined as a diagnosis in the National Patient Registry (ICD‐10 code: F00, G30) or initiation of pharmacological treatment for Alzheimer's disease (ATC‐code: N06D); new‐onset of vascular dementia in the National patient registry (ICD‐10 code: F01), Korsakoff's syndrome, or alcohol dementia in the National Patient Registry (ICD‐10 code: F10.6 or F10.7).

### 2.3 Matching

The matching procedure was conducted in two stages. First, study cohorts were defined according to index date. For individuals undergoing MBS, the index date was the day of surgery. For individuals in the non‐operated group, the index date was defined as the status update closest to July 2 of the corresponding calendar year. Eligibility assessment and matching were performed separately for each calendar year to ensure comparability. Each individual could only be included once in the study.

In the second stage, a 1:1–2 propensity score matching with a generalized linear model and a caliper of 0.2 was performed. Based on previously reported risk factors for dementia,^3^, ^20^ the model included the following variables: age, BMI at the index date, sex, hypertension, dyslipidemia, cardiovascular comorbidity, chronic obstructive pulmonary disease (COPD), depression, previous alcohol use disorder, diabetes status (glycated hemaglobin A1c [HbA1c], type of diabetes treatment), smoking status, disposable income, level of education, marital status, and area of residence as variables in the model. A separate match was performed for each calendar year to ensure adequate matching on the status of that individual index year. A second matching procedure was performed with a 1:5 exact match (on age, sex, and area of residence) with the surgery group to a normal population to estimate the residual risk in patients with obesity and T2D following bariatric surgery relative to the background population risk. Matched controls with previous dementia diagnosis, emigration, or mortality (up to 1 year after the index date), previous diagnosis of cerebrovascular events, brain cancer, or pharmacological treatment for Parkinson's disease or parkinsonism were excluded.

A sensitivity analysis with a similar method was performed, including patients in the surgical group who died, emigrated, or were diagnosed with dementia during the first year after surgery.

### 2.4 Statistics

Categorical variables are presented as *n* (%). Continuous variables assuming normal distribution are presented as mean (standard deviation), and variables not assuming normal distribution as median (interquartile range). Standardized mean differences (SMD) were calculated for all baseline variables to assess balance between groups, with an SMD > 0.1 being considered to represent a clinically meaningful remaining imbalance. The SMD for continuous variables not assuming normal distribution was adjusted according to Hedges and Olkin.^21^

Risk for dementia was evaluated using a Fine–Gray subdistribution hazard model, implemented as a weighted Cox regression on the subdistribution risk set using inverse probability weights, with cluster‐robust standard errors to preserve the matched pair structure and subdistribution hazard ratios (SHR) and 95% confidence intervals (95% confidence interval [CI]) as measures of association.^22^, ^23^ The model was adjusted for variables with remaining imbalance (BMI, smoking status, and level of education) and for variables showing borderline imbalance (type of T2D treatment). Patients were followed until death, migration, event, or December 31, 2024, whichever came first. Time to event was estimated with the Kaplan‐Meier method and visualized as cumulative probability (1‐ Kaplan–Meier estimate). A second sensitivity analysis was performed to address missing data in smoking status and level of education with imputed using logistic regression and level of education using multinomial logistic regression, with all model covariates and auxiliary variables (age, sex, hypertension, depression, cardiovascular comorbidity, dyslipidemia, COPD, previous alcohol use disorder, HbA1c, and disposable income) included as predictors. Outcome and competing event variables were excluded from the imputation predictor matrix to avoid the use of future information. The Fine–Gray subdistribution hazard model with cluster‐robust standard errors was fitted in each of the five imputed datasets and results were pooled using Rubin's rules.^24^

For the comparison between the surgical group and the normal population control group, a Fine–Gray subdistribution hazard model with cluster‐robust standard errors was fitted for each outcome, with death as the competing event. No covariate adjustment was performed in this comparison as the groups were matched on age, sex, and county of residence.

The proportional hazards assumption was assessed for all Cox models using Schoenfeld residuals and the scaled Schoenfeld residuals test.^25^ The cumulative probability (1 ‐ Kaplan–Meier estimate) was used to visualize the unadjusted event‐free survival for each outcome. Cumulative incidence functions accounting for the competing risk of death was additionally estimated and presented graphically.

SPSS version 29 (IBM, Armonk, NY, USA), Stata version 17.0 (StataCorp, College Station, TX, USA), and R Studio version 2026.01.0 (R Foundation for Statistical Computing, Vienna, Austria) were used for statistical analyses.

### 2.5 Ethics

The study was approved by the National Research Ethics Committee, and performed in accordance with the ethical standards as laid down in the 1964 Declaration of Helsinki and its later amendments.

## 3 RESULTS

From January 2007 until December 2023, 12,251 patients with T2D and obesity meeting the inclusion criteria underwent a primary gastric bypass or sleeve gastrectomy procedure. An additional 334,492 patients with T2D and obesity who did not undergo bariatric surgery were identified from the NDR. After propensity score matching, 12,084 remained in the surgery group, and 23,581 in the matched non‐operated group. During the follow‐up period, 924 patients in the surgery group died, and 120 patients emigrated. In the non‐operated group, 2479 died, and 220 patients emigrated. Mean follow‐up time was 9.5 ± 4.37 years in the surgical group and 9.2 ± 4.36 years in the non‐surgical group. In total, 1153 patients (9.5%) in the surgical group and 13487 (57.2%) in the non‐surgical group received treatment with a GLP‐1RA for at least 1 year during the study period.

The two groups were well matched, except for a remaining imbalance for BMI, smoking status, and level of education (with an SMD > 0.1; Table 1).

**TABLE 1:** Baseline characteristics of the matched cohort of patients with T2D and obesity.

Parameter	Surgery group	Non‐surgical group	Standardized mean difference
n	12084	23581	—
Age, years	48.2 ± 9.87	48.5 ± 11.77	0.028
Body mass index, kg/m^2^	41.9 ± 5.59	41.2 ± 6.79	0.113
**Sex**			
Men	4696 (38.9%)	9252 (39.2%)	0.006
Women	7388 (61.1%)	14329 (60.8%)	0.006
**Comorbidities**			
Hypertension	7806 (64.6%)	15406 (65.3%)	0.015
Dyslipidemia	5287 (43.8%)	10450 (44.3%)	0.010
Cardiovascular comorbidity	1351 (11.2%)	2494 (10.6%)	0.019
Chronic obstructive pulmonary disorder	522 (4.3%)	983 (4.2%)	0.005
Depression	5002 (41.4%)	9825 (41.7%)	0.006
Alcohol use disorder	410 (3.4%)	820 (3.5%)	0.005
**Diabetes status**			
HbA1c, mmol/mol	57.6 ± 16.29	57.7 ± 16.88	0.006
**Type of treatment**			
Non‐pharmacological	2709 (22.4%)	4638 (19.7%)	0.066
Non‐insulin pharmacological treatment	6342 (52.5%)	13516 (57.3%)	0.097
Insulin	3033 (25.1%)	5427 (23.0%)	0.049
History of smoking^a^	4516 (38.0%)	6974 (31.3%)	0.141
Disposable income, thousand SEK	283.8 (200.4–376.5)	263.3 (182.8–362.3)	0.085
**Education** ^b^			
Primary (9 years)	2329 (19.3%)	4785 (20.3%)	0.025
Secondary (10–12 years)	6916 (57.2%)	12107 (51.3%)	0.119
Higher (12 years)	2753 (22.8%)	6277 (26.6%)	0.088
**Marital status**			
Single	5440 (45.0%)	10640 (45.1%)	0.002
Living with spouse	6644 (55.0%)	12941 (54.9%)	0.002
**Residential area**			
Large city	4165 (34.5%)	8126 (34.5%)	0.000
Medium‐size city	4722 (39.1%)	9488 (40.2%)	0.022
Rural	3197 (26.5%)	5967 (25.3%)	0.027

*Note*: Mean standard deviation for continuous values assuming normal distribution and median (interquartile range) for values not assuming normal distribution. Categorical values are presented as numbers (percentages).

^a^ Missing information for 197 patients in the surgery group (1.6%), and 1303 (5.5%) in the non‐surgical group.

^b^ Missing information for 86 patients in the surgical group (0.7%), and 412 (1.7%) in the non‐surgical group.

### 3.1 Operation data

The surgical procedures were RYGB in 10,137 patients, SG in 1941 patients, and single‐anastomosis gastric bypass in 6 patients. Median operating time was 64 min (interquartile range [IQR] 46–88 min). Median postoperative length of stay was 1 day (IQR 1–2 days). Follow‐up at day 30 for postoperative complications were registered for 11,945 patients (98.8%), of whom 1088 (9.1%) suffered from a postoperative complication of any form. A serious postoperative complication occurred within 30 days after 391 operations (3.3%).

### 3.2 BMI trajectories

**FIGURE 1:**
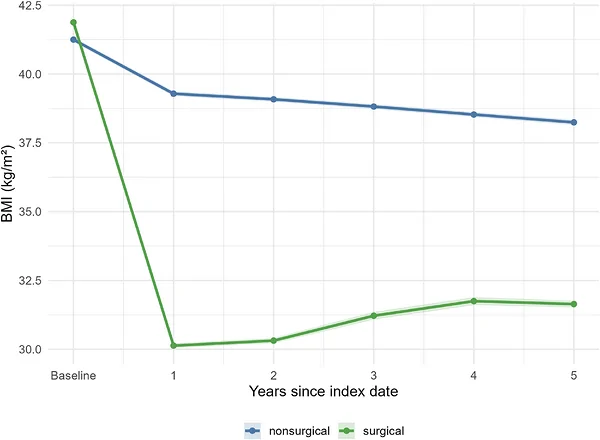
BMI trajectories for patients with obesity and T2D who undergo surgery or not up to 5 years after index date. Lines represent mean BMI, and shaded areas represent 95% confidence intervals. BMI, body mass index; T2D, type 2 diabetes.

The lowest BMI in the surgery group was seen at 1 year after surgery, followed by a slight increase over time. At 5 years after surgery, mean BMI was 31.6 ± 5.33 in the surgical group and 38.3 ± 6.44 in the non‐surgical group (*p* < 0.001; Figure 1).

### 3.3 Risk for dementia

**FIGURE 2:**
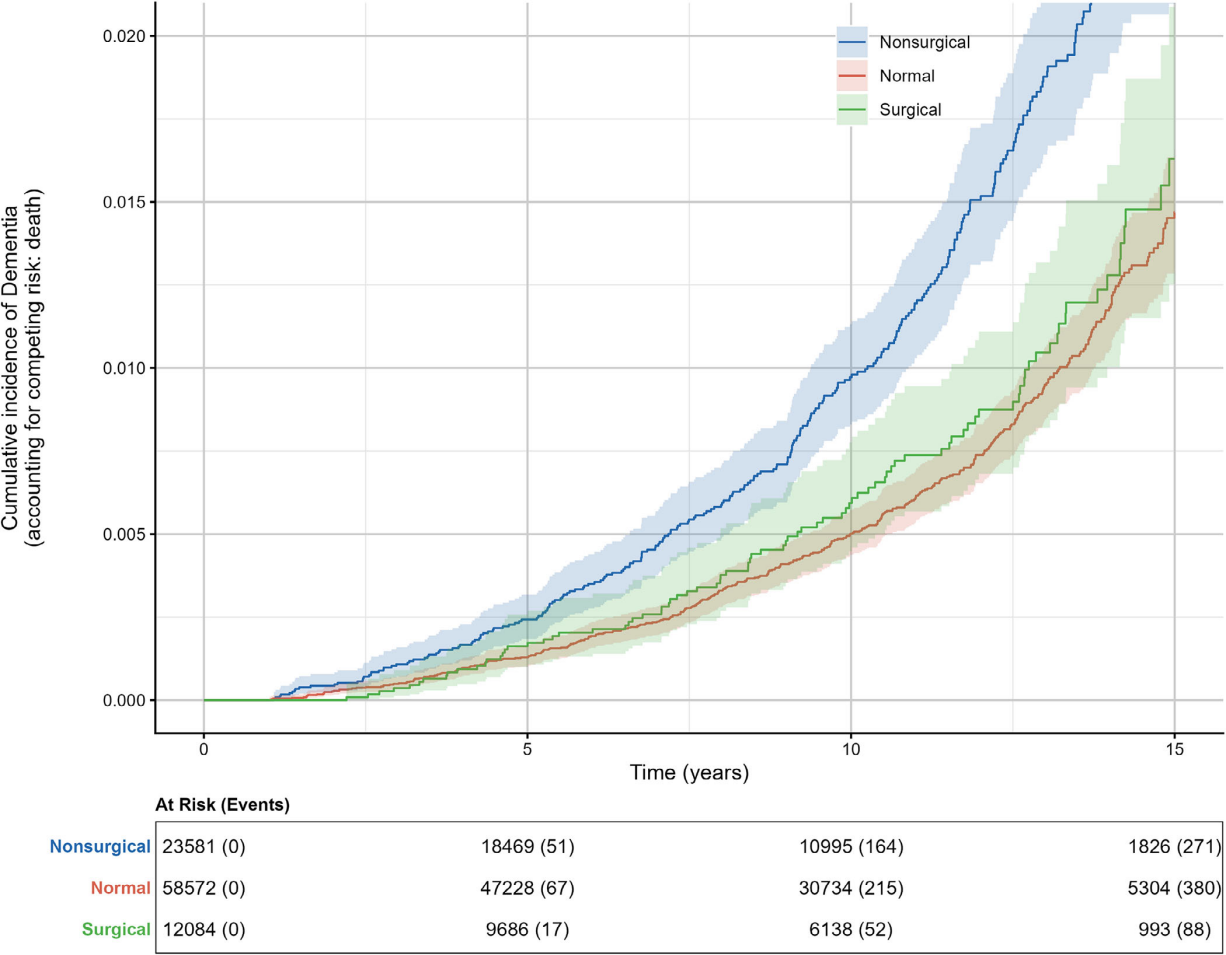
Risk for new onset of all‐cause dementia (1‐ Kaplan–Meier estimate) for patients with obesity and T2D who undergo surgery or not, and controls (age, sex, and area of residence) from the normal population. T2D, type 2 diabetes.

Overall, 91 patients were diagnosed with dementia after surgery (15‐year cumulative incidence 1.8%), and 283 in the non‐surgical group (15‐year cumulative incidence 2.7%). Surgery was associated with a lower risk for new onset of all‐cause dementia when compared to patients in the non‐operated group (SHR 0.60, 95%CI 0.47–0.76, *p* < 0.001; Table 2; Figure 2), but no difference when compared to the normal population (SHR 1.12, 95%CI 0.89–1.41, *p* = 0.330). A sensitivity analysis including patients in the surgery group who were excluded during the first year showed similar results (Tables S2 and S3).

**TABLE 2:** Risk for dementia comparing patients with obesity and T2D who underwent MBS to matched controls who did not undergo surgery.

	Fine–Gray cluster^a^		Sensitivity analyses^b^	
Parameter	SHR (95%CI)	*p*‐value	SHR (95%CI)	*p*‐value
All‐cause dementia	0.60 (0.47–0.76)	< 0.001	0.61 (0.48–0.77)	0.005
Alzheimer's disease	0.56 (0.41–0.76)	< 0.001	0.56 (0.41–0.76)	0.008
Vascular dementia	0.49 (0.29–0.85)	0.011	0.48 (0.28–0.82)	0.032
Alcohol‐related dementia	3.67 (1.33–10.14)	0.012	3.83 (1.35–10.84)	0.041

Abbreviations: BMI, body mass index; CI, confidence interval; SHR, subdistribution hazard ratio.

^a^ Fine–Gray subdistribution hazard model using weighted Cox approach, adjusted for BMI, smoking status, level of education and type of diabetes treatment, with cluster‐robust standard errors accounting for matched pair correlation and death as the competing event.

^b^ As model 1, with missing values in smoking status and level of education imputed using multiple imputation by chained equations (*m* = 5); results pooled using Rubin's rules.

### 3.4 Risk for Alzheimer's disease

**FIGURE 3:**
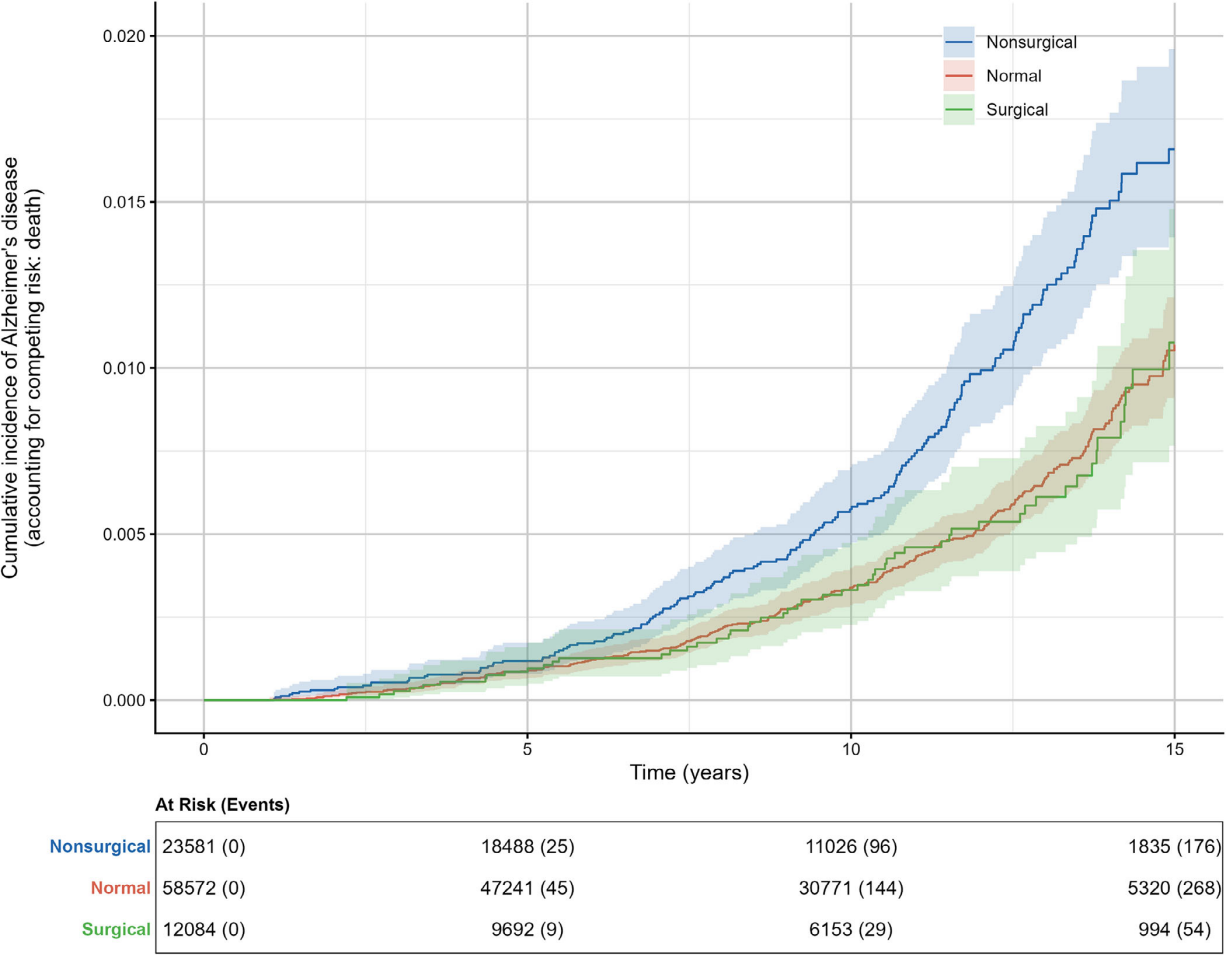
Risk for new onset of Alzheimer's disease (1‐ Kaplan–Meier estimate) for patients with obesity and T2D who undergo surgery or not, and controls (age, sex, and area of residence) from the normal population. T2D, type 2 diabetes.

Alzheimer's disease was diagnosed in 54 patients after surgery (15‐year cumulative incidence 1.2%) and 185 patients in the non‐surgical group (15‐year cumulative incidence 1.9%). The surgical group had a lower risk for new onset of Alzheimer's disease when compared to patients in the non‐operated group (SHR 0.56, 95%CI 0.41–0.76, *p* < 0.001; Table 2; Figure 3), but similar to that seen in the normal population (HR 0.97, 95%CI 0.73–1.30, *p* = 0.855).

### 3.5 Risk for vascular dementia

Vascular dementia diagnosis was registered for 17 patients after surgery (15‐year cumulative incidence 0.4%) and 65 patients in the non‐surgical group (15‐year cumulative incidence 0.7%). Surgery was associated with a lower risk for new onset of vascular dementia when compared to the non‐operated group (SHR 0.49, 95%CI 0.29–0.85, *p* = 0.011; Table 2; Figure S1) but a higher risk remained when compared to the normal population (HR 2.67, 95%CI 1.44–4.95, *p* = 0.002).

### 3.6 Risk for Korsakoff's syndrome and alcohol‐related dementia

Korsakoff's syndrome or alcohol‐related dementia was registered for 10 patients after surgery (15‐year cumulative incidence 0.2%) and 6 patients in the non‐surgical group (15‐year cumulative incidence 0.04%). The risk was higher for the surgical group when compared to the non‐operated group (SHR 3.67, 95%CI 1.33–10.14, *p* = 0.012; Table 2; Figure S2), but no difference was seen when compared to the normal population (SHR 1.55, 95%CI 0.73–3.29, *p* = 0.251).

## 4 DISCUSSION

MBS was associated with a reduced risk of Alzheimer's disease and vascular dementia, but an increased risk of alcohol‐related dementia, among patients with obesity and T2D compared with those who did not undergo MBS. The risk of Alzheimer's disease following MBS approached that of the normal population, whereas the risk of vascular dementia remained elevated relative to the normal population.

Obesity is associated with several potential mechanisms of dementia. These include inducing inflammatory mediators that can affect the central nervous system via the blood‐brain barrier, elevated cortisol, changes in microbiota, and changes in adipocyte dysfunction.^6^ Weight reduction and improved glycemic control are key therapeutic targets in individuals at risk for dementia since they are associated with a reduction in inflammatory markers. Although the observed effects are likely largely attributable to weight loss, non‐weight‐dependent mechanisms may also contribute, as has been previously described.^26^, ^27^ MBS induces changes in gut‐derived hormones, including GLP‐1, and has been reported to activate GLP‐1/SGLT1 signaling pathways in the hippocampus, potentially reversing nerve degenerative processes.^28^ Pharmacological treatment with GLP‐1RAs mimics aspects of these mechanisms and has been proposed to confer neuroprotective effects, with real‐world studies suggesting up to a 70% reduction in dementia incidence among long‐term users.^29^, ^30^ However, in a randomized clinical trial, oral semaglutide did not slow cognitive decline in individuals with early Alzheimer's disease, suggesting that such therapies may be more effective for prevention than for treatment.^31^ Whether similar neuroprotective effects can be attributed to MBS remain uncertain and were beyond the scope of the current study. Although a substantial proportion of patients in this study received GLP‐1RA therapy, the most effective agents, that is, semaglutide and tirzepatide, have only been available in Sweden since 2018 and 2024, respectively. Therefore, a meaningful subgroup analysis comparing MBS to GLP‐1RA treatment was not feasible. Furthermore, real‐world studies have suggested greater risk reduction for several diabetes‐related complications, including cardiovascular events following MBS^32^, ^33^ compared to treatment with GLP‐1RA. Therefore, the findings of the present study cannot be directly extrapolated to pharmacological treatments targeting the gut‐brain axis.

MBS was associated with an increased risk of alcohol‐related dementia. Alcohol overconsumption remains a challenging long‐term complication of MBS, with some evidence of a higher incidence after RYGB compared with SG.^34^ In addition, MBS have been associated with risks of clinical depression, malnutrition, and micronutrient deficiencies.^35^ Social isolation, although affecting a minority of patients, may also occur postoperatively.^36^ Collectively, these factors may contribute to an increased risk of dementia and could explain the higher incidence of Korsakoff's syndrome and alcohol‐related dementia observed in this study. Although the absolute incidence of these conditions remained low, these findings underscore the importance of long‐term follow‐up and multidisciplinary support for patients undergoing MBS, with particular attention to surgical and non‐surgical complications, mental health, and adherence to postoperative recommendations (including supplementation).

The present study was limited to patients with obesity and T2D. In parallel with the global obesity pandemic, the prevalence of T2D continues to rise, and an increasing proportion of newly diagnosed individuals living with obesity.^37^ People with obesity and T2D have particularly substantial benefits from MBS^38^ and should be considered for surgical intervention at an early stage following diagnoses, as the likelihood of remission appears to decline with longer disease duration.^39^ However, the exclusion of patients without T2D limits the generalizability of these findings to patients without T2D. Additionally, the study was conducted within a publicly funded Scandinavian healthcare system and a predominantly Caucasian population, which may further limit generalizability to other settings and populations. Finally, 84% of the patients in the surgical group underwent RYGB. With previous studies suggesting differences in surgical outcomes after RYGB compared to SG beyond differences in weight‐related outcomes, ^26^ the results of the current study may therefore not necessarily be generalizable to other MBS procedures than RYGB.

The major strengths of this study include its nationwide design and the high inclusion rate of patients within the defined cohorts. The use of unique personal identification numbers enabled linkage to comprehensive national registries, allowing access to detailed data on specialized care, prescribed drugs, socioeconomic factors, and mortality. Nevertheless, the findings must be interpreted in light of the study's limitations. Although the definition of dementia has been previously validated with high validity for dementia overall, the validity for specific causes of dementia (in particular unspecified dementia) remains lower.^19^ While the definition was strengthened by the inclusion of pharmacological treatment for Alzheimer's disease, diagnostic delay remains a potential concern.^40^ Further, early personality traits associated with the development of dementia may reduce the probability of seeking surgical obesity treatment, introducing a risk for confounding by indication. To mitigate reverse causation, a 12‐month washout period was applied. The Kaplan–Meier curve showed an apparent increasing risk reduction over time, further supporting the plausibility of the findings. While the propensity score match was generally well matched, imbalance remained for BMI, smoking status, and level of education in favor of the non‐operated group. The variables were further adjusted for in the statistical analyses reducing the risk for a significant impact on the outcomes. Several established risk factors for dementia were accounted for in the study; others–such as prior traumatic brain injury, air pollution exposure, and physical inactivity–were not available. Including city size in the matched model may partially account for environmental exposures such as air pollution, and there is no indication of differential bias between groups with respect to these factors.

In conclusion, among patients with obesity and T2D, MBS was associated with a reduced risk of Alzheimer's disease and vascular dementia, but an increased risk of alcohol‐related dementia. Overall, MBS was associated with a lower risk of all‐cause dementia in this population.

## CONFLICT OF INTEREST STATEMENT

E.S. has received consultant fees from Johnson & Johnson Medical (to the institution) for work unrelated to the present manuscript. None of the remaining authors declares any conflict of interest. Author disclosures are available in the Supporting Information.

## CONSENT STATEMENT

All patients were informed of the clinical registries and that registered data would be subject to clinical studies. They were at all times free to reject inclusion in these registries (“opt‐out”). Informed consent for this particular study was waived after evaluation and approval by the National Ethics Committee in Sweden.

## ROLE OF THE FUNDING SOURCE

The funders had no role in the design and conduct of the study; collection, management, analysis, and interpretation of the data; preparation, review, or approval of the manuscript; and decision to submit the manuscript for publication.

## Supporting information



Supporting Information

Supporting Information

## Data Availability

Data cannot be shared publicly because of patient confidentiality under current Swedish legislation. Data are available from the Scandinavian Obesity Surgery Registry (contact via soreg@regionorebrolan.se), the Swedish Board of Health and Welfare (contact via Registerservice@socialstyrelsen.se), and Statistics Sweden (contact via mikrodata@scb.se) for researchers who meet the criteria for access to confidential data.
