# Supplementary material for: The effects of bariatric surgery on dementia in patients with diabetes and obesity
Source: Alzheimers Dement. 2026 Aug 21;22(8):e71802. doi: 10.1002/alz.71802 (PMC13498901; doi:10.1002/alz.71802)
Supplement: Supplementary file 2 [file ALZ-22-e71802-s001.docx]

**The effects of bariatric surgery on dementia in patients with diabetes and obesity**

Erik Stenberg MD, PhD^1^; Yang Cao PhD^2,3^; Björn Eliason MD, PhD^4^; Erik Näslund MD, PhD^5^

Affiliation:

1. Department of Surgery, Faculty of Medicine and Health, Örebro University, Örebro, Sweden
2. Clinical Epidemiology and Biostatistics, School of Medical Sciences, Faculty of Medicine and Health, Örebro University, Örebro, Sweden
3. Unit of Integrative Epidemiology, Institute of Environmental Medicine, Karolinska Institutet, Stockholm, Sweden
4. Department of Medicine, Sahlgrenska University Hospital, Gothenburg, Sweden
5. Division of Surgery, Department of Clinical Sciences, Danderyd Hospital, Karolinska Institutet, Stockholm, Sweden

| Table of contents | |
| --- | --- |
| SAP - Statistical analysis plan | Page 2 |
| Table S1. Definition of baseline variables | Page 4 |
| Table S2. Baseline characteristics of the matched cohort of patients with type-2 diabetes and obesity after including patients in the surgery cohort with mortality, dementia diagnosis and emigration within the first year after surgery | Page 5 |
| Table S3. Risk for dementia comparing patients with obesity and type-2 diabetes who underwent metabolic and bariatric surgery to matched controls who did not undergo surgery, including patients in the surgery cohort with mortality, dementia diagnosis, and emigration within the first year after surgery | Page 6 |
| Fig S1. Risk for new onset of vascular dementia | Page 7 |
| Fig S2. Risk for new onset of alcohol dementia | Page 8 |

**Statistical analysis plan**

**Setting**

Health care in Sweden is tax-funded, providing equal access to all residents. A unique personal identity number (PIN) is assigned to all residents, which enables linkage of nationwide registers (Table S1).

**Study cohorts**

*Bariatric surgery cohort*

The bariatric surgery (BS) cohort includes adults (≥18 to <75 years) with a diagnosis of type-2 diabetes and obesity (BMI≥30) undergoing primary gastric bypass or sleeve gastrectomy procedure between 2007 and 2023, registered in the Scandinavian Obesity Surgery Registry, SOReg.

*Non-bariatric surgery cohort*

The non-bariatric surgery (NBS) cohort consists of patients with type-2 diabetes (age ≥18 to <75 years and BMI≥30) who had not undergone bariatric surgery before 2024.

*Exclusion criteria*

Exclusion criteria are: Pharmacological treatment or diagnosis of dementia up to 1 year after the inclusion date. Cerebrovascular event, brain cancer, or pharmacological treatment for Parkinson’s disease or parkinsonism at any time before the index date.

**Matching**

Individuals in the BS cohort and the NBS cohort will be matched 1:2 using Propensity Score (PS) matching, nearest function with a caliper of 0.2 including the following variables: age, BMI, sex, hypertension, dyslipidemia, cardiovascular comorbidity, chronic obstructive pulmonary disease (COPD), depression, previous alcohol use disorder, diabetes status (HbA1c, type of diabetes treatment), smoking status, disposable income, level of education, marital status and area of residence.

The index date is defined as the day of surgery in the BS group. A separate match will be conducted for each year to allow an appropriate match to the status at that time for the NBS group.

A second match (exact) based on age, sex, and area of residence will be conducted to compare the results to that of a representative group from the normal population. The same exclusion criteria that apply to the study group will also be applied to this group.

**Outcomes**

*Primary outcome:*

Diagnosis or pharmacological treatment for dementia

*Secondary outcomes:*

-Alzheimer’s disease

-Vascular dementia

-Korsakoff’s syndrome or alcohol dementia

Statistical analysis plan

The two groups will be matched using Propensity score matching (nearest function) with a caliper of 0.2 including age, BMI, sex, hypertension, dyslipidemia, cardiovascular comorbidity, chronic obstructive pulmonary disease (COPD), depression, previous alcohol use disorder, diabetes status (HbA1c, type of diabetes treatment), smoking status, disposable income, level of education, marital status and area of residence. A standardized mean difference of ≥0.1 will be considered residual imbalance and adjusted for in further statistical analyses.

Continuous variables assuming normal distribution will be presented as means +/- standard deviation. Variables not assuming normal distribution will be presented as medians with interquartile ranges (IQR). Categorical variables will be presented as numbers and proportions (%).

Risk for the main and secondary outcomes will be evaluated using a Fine-Gray subdistribution hazard model, implemented as a weighted Cox regression on the subdistribution risk set using inverse probability weights, with cluster-robust standard errors to preserve the matched pair structure and subdistribution Hazard Ratios (SHR) and 95% confidence intervals (95% CI) as measures of association. The model will be adjusted for variables with remaining imbalance. Patients will be followed until death, migration, event, or end of follow-up. Time to event was estimated with the Kaplan-Meier method and visualized as cumulative probability (1- Kaplan-Meier estimate).

In case of missing data, sensitivity analysis will be performed following imputed datasets, and results will be pooled using Rubin's rules (fitted within 5 imputed data sets).

| **Table S1.** Definition of baseline variables | | |
| --- | --- | --- |
| Variable | Source | Definition |
| Body Mass Index | SOReg, NDR | Based on reported BMI before preoperative weight reduction (surgery group), or at the index date (non-surgery group). Follow-up date was based on information from SOReg and NDR |
| Hypertension | Prescribed drugs registry | Pharmacological treatment within 12 months before the index date (ATC-code: C02, C03, C07, C08, C09) |
| Dyslipidemia | Prescribed drugs registry | Pharmacological treatment within 12 months before the index date (ATC-code: C10) |
| Cardiovascular comorbidity | National Patient registry | Specialized care for ischemic heart disease (I20-I22), arrhythmic heart disease (I47-I48), or heart failure (I50) |
| Chronic obstructive pulmonary disorder | National Patient registry, Prescribed drugs registry | Hospitalization for COPD or a complication of COPD with COPD as secondary diagnosis (ICD-10: J44), or prescription of an anticolinergic drug (ATC-code:R03BB), long-acting beta-2 antagonist (ATC-codes: R03AC12-R03AC18), or a combination of these (ATC-code: R03AL) |
| Depression | Prescribed drugs registry | ATC-code: N06A |
| Alcohol use disorder | National Patient registry, Prescribed drugs registry | Specialized care for mental or behavioral disorders due to alcohol use (ICD-10: F10), or treatment for alcohol use disorder (ATC-code: N07BB) at any time before index date. |
| HbA1c, mmol/mol | SOReg, NDR | Based on reported BMI before preoperative weight reduction (surgery group), or at the index date (non-surgery group) |
| Type of diabetes treatment | Prescribed drugs registry | Type of treatment in the year before the index date |
| Smoking status | SOReg, NDR | History of smoking, or no history of smoking reported up until the index date. |
| Disposable income, thousand SEK | Statistics Sweden (Longitudinal integrated database for health insurance, labour market studies) | Individual reported information on income per consumer unit. Defined as the disposable household income (after taxes and negative transfers) divided by the household consumption weight (in accordance with current Swedish standards as defined by Statistics Sweden); adjusted to the 2024 consumer prize index. |
| Education | Statistics Sweden (Longitudinal integrated database for health insurance, labour market studies) | The highest completed education at the index date. |
| Marital status | Statistics Sweden (Longitudinal integrated database for health insurance, labour market studies) | Defined as single, or living with a spouse |
| Residential area | Statistics Sweden | Type of residential town at the index date. |

| **Table S2.** Baseline characteristics of the matched cohort of patients with type-2 diabetes and obesity after including patients in the surgery cohort with mortality, dementia diagnosis and emigration within the first year after surgery | | | |
| --- | --- | --- | --- |
|  | Surgery group | Non-surgical group | Standardized mean difference |
| n | 12152 | 23720 | - |
| Age, years | 48.2 ± 9.87 | 48.5 ± 11.77 | 0.028 |
| Body Mass Index, kg/m^2^ | 41.9 ± 5.59 | 41.2 ± 6.79 | 0.113 |
| Sex |  |  |  |
| Men | 4732 (38.9%) | 9390 (39.6%) | 0.013 |
| Women | 7420 (61.1%) | 14330 (60.4%) | 0.013 |
| Comorbidities |  |  |  |
| Hypertension | 7847 (64.6%) | 15447 (65.1%) | 0.011 |
| Dyslipidemia | 5323 (43.8%) | 10588 (44.6%) | 0.017 |
| Cardiovascular comorbidity | 1371 (11.3%) | 2603 (11.0%) | 0.010 |
| Chronic obstructive pulmonary disorder | 528 (4.3%) | 1049 (4.4%) | 0.004 |
| Depression | 5007 (41.2%) | 9777 (41.2%) | 0.000 |
| Alcohol use disorder | 418 (3.4%) | 844 (3.6%) | 0.006 |
| Diabetes status |  |  |  |
| HbA1c, mmol/mol | 57.6 ± 16.29 | 57.7 ± 16.88 | 0.003 |
| Type of treatment |  |  |  |
| Non-pharmacological | 2717 (22.4%) | 4695 (19.8%) | 0.063 |
| Non-insulin pharmacological treatment | 6374 (52.5%) | 13627 (57.4%) | 0.101 |
| Insulin | 3061 (25.2%) | 5398 (22.8%) | 0.057 |
| History of smoking^1^ | 4531 (37.9%) | 6833 (30.6%) | 0.155 |
| Disposable income, thousand SEK | 283.8 (200.3-376.1) | 264.9 (182.6-363.5) | 0.078 |
| Education^2^ |  |  |  |
| Primary (9 years) | 2347 (19.5%) | 4772 (20.5%) | 0.026 |
| Secondary (10-12 years) | 6948 (57.6%) | 12282 (52.7%) | 0.119 |
| Higher (12 years) | 2767 (22.9%) | 6245 (26.8%) | 0.098 |
| Marital status |  |  |  |
| Single | 5479 (45.1%) | 10667 (45.0%) | 0.002 |
| Living with spouse | 6673 (54.9%) | 13053 (55.0%) | 0.002 |
| Residential area |  |  |  |
| Large city | 4186 (34.4%) | 8111 (34.2%) | 0.005 |
| Medium-size city | 4745 (39.0%) | 9509 (40.1%) | 0.021 |
| Rural | 3221 (26.5%) | 6100 (25.7%) | 0.018 |

Mean standard deviation for continuous values assuming normal distribution and median (interquartile range) for values not assuming normal distribution. Categorical values are presented as numbers (percentage).

1- Missing information for 207 patients in the surgery group (1.7%), and 1383 (5.8%) in the non-surgical group
2- Missing information for 90 patients in the surgical group (0.7%), and 421 (1.8%) in the non-surgical group

| **Table S3**. Risk for dementia comparing patients with obesity and type-2 diabetes who underwent metabolic and bariatric surgery to matched controls who did not undergo surgery, including patients in the surgery cohort with mortality, dementia diagnosis, and emigration within the first year after surgery | | | | |
| --- | --- | --- | --- | --- |
|  | Fine-Grey cluster^1^ | | Sensitivity analyses^2^ | |
|  | SHR (95%CI) | P | SHR (95%CI) | P |
| All-cause dementia | 0.59 (0.46-0.74) | <0.001 | 0.59 (0.46-0.74) | 0.003 |
| Alzheimer’s disease | 0.54 (0.40-0.74) | <0.001 | 0.55 (0.40-0.74) | 0.006 |
| Vascular dementia | 0.51 (0.30-0.89) | 0.016 | 0.51 (0.30-0.87) | 0.045 |
| Alcohol-related dementia | 2.96 (1.02-8.55) | 0.046 | 3.11 (1.12-8.65) | 0.041 |

SHR = Subdistribution Hazard ratio; CI = confidence interval

1. Fine-Gray subdistribution hazard model using weighted Cox approach, adjusted for BMI, smoking status, level of education and type of diabetes treatment, with cluster-robust standard errors accounting for matched pair correlation and death as the competing event.

2. As model 1, with missing values in smoking status and level of education imputed using multiple imputation by chained equations (m=5); results pooled using Rubin's rules. MI, multiple imputation.

*Fig S1. Risk for new onset of vascular dementia (1-Kaplan-Meier estimate) for patients with obesity and type-2 diabetes who undergo surgery or not, and controls (age,sex and area of residence) from the normal population.*

*Fig S2. Risk for new onset of alcohol dementia (1-Kaplan-Meier estimate) for patients with obesity and type-2 diabetes who undergo surgery or not, and controls (age,sex and area of residence) from the normal population.*
